# Three-Dimensional Fabrication for Microfluidics by Conventional Techniques and Equipment Used in Mass Production

**DOI:** 10.3390/mi7050082

**Published:** 2016-05-04

**Authors:** Toyohiro Naito, Makoto Nakamura, Noritada Kaji, Takuya Kubo, Yoshinobu Baba, Koji Otsuka

**Affiliations:** 1 Department of Material Chemistry, Graduate School of Engineering, Kyoto University, Katsura, Nishikyo-ku, Kyoto 615-8510, Japan; nakamura.makoto.87a@st.kyoto-u.ac.jp (M.N.); kubo@anchem.mc.kyoto-u.ac.jp (T.K.); otsuka@anchem.mc.kyoto-u.ac.jp (K.O.); 2 Department of Applied Chemistry, Graduate School of Engineering, Nagoya University, Furo-cho, Chikusa-ku, Nagoya 464-8603, Japan; kaji@apchem.nagoya-u.ac.jp (N.K.); babaymtt@apchem.nagoya-u.ac.jp (Y.B.); 3 ImPACT Research Center for Advanced Nanobiodevices, Nagoya University, Furo-cho, Chikusa-ku, Nagoya 464-8603, Japan

**Keywords:** 3D microfluidics, microfabrication

## Abstract

This paper presents a simple three-dimensional (3D) fabrication method based on soft lithography techniques and laminated object manufacturing. The method can create 3D structures that have undercuts with general machines for mass production and laboratory scale prototyping. The minimum layer thickness of the method is at least 4 µm and bonding strength between layers is over 330 kPa. The performance reaches conventional fabrication techniques used for two-dimensionally (2D)-designed microfluidic devices. We fabricated some 3D structures, *i.e.*, fractal structures, spiral structures, and a channel-in-channel structure, in microfluidic channels and demonstrated 3D microfluidics. The fabrication method can be achieved with a simple black light for bio-molecule detection; thus, it is useful for not only lab-scale rapid prototyping, but also for commercial manufacturing.

## 1. Introduction

Research in the field of microfluidics is moving from understanding two-dimensionally (2D)-designed flow to three-dimensionally (3D)-designed flow. 2D flow, which is planar flow with no vertical flow, has been studied since the 1990s, and various 2D-designed microfluidic devices such as multiple branching channels [1,2,3], zigzag shaped channels [3,4], Tesla mixers [5], and deterministic lateral displacement devices [6,7,8] have been developed. These channels have no changes in their cross-sectional shape and are fabricated by simple soft lithography or an etching process. In some fairly recent reports, Dean flow which is 3D flow formed in a 2D curved microchannel has been applied for particle separation [9,10] and liquid mixing [11,12]. To generate more complex 3D flows in microchannels, 3D-designed structures with configuration changes in a vertical direction have been developed since about 2000. The chaotic mixer reported in 2002 has structures that are grooves similar to rifling in a gun barrel; the structures can generate a 3D twisting flow in the mixer channel [13,14]. The microfluidic baker’s transformation (MBT) device that has 2D-designed structures with vertical changes also generate a 3D flow in a microchannel [15,16]. These 3D structures achieved a highly efficient solution mixing in microfluidic channels. However, their configurations were limited by the demolding process to shapes without undercuts such as cuboids or pyramids. Most reports have used 2D or 3D structures without undercuts for microfluidic devices in spite of the research shift from understanding 2D flow to 3D flow due to the difficulties and particularities of the fabrication processes of realizing complex 3D structures unlimited by the demolding process.

To fabricate complex 3D structures with undercuts, 3D microfabrication techniques are required. The complex 3D structures can be fabricated using additive manufacturing such as microstereolithography [17,18] or lamination [19,20]. Microstereolithography employs a liquid UV-curable polymer, an UV laser, and an XYZ stage to build thin layers which are part of a 3D structure. The UV laser patterns the cross sections of a 3D structure on a thin layer with a curing UV-curable polymer. Subsequently, the XYZ stage moves down to the next layer. These two processes are repeated over and over for creating a 3D structure. Although microstereolithography can achieve complex 3D structures, there are some problems: (i) it needs special equipment; (ii) a large amount of UV-curable polymer compared with microstructure is required; and (iii) the method limits the fabrication speed and working area because of the laser scanning method. These are critical issues for the progress of the technology from the basic research phase to the market-related development phase.

Lamination of many thin layers of substrates is a widely used method thanks to the use of conventional fabrication techniques and machines [20,21,22,23]. Although this method is possible for the mass production of micro 3D structures, two drawbacks have to be solved. One is that some materials require a cumbersome bonding process to build up layers with chemical, thermal, or both processes. The other is the difficulty of controlling the layer thickness. The layer thickness of the lamination method is not thin enough for creating 3D structures in microfluidic channels.

Many 3D printers based on microstereolithography or lamination techniques have been launched in the last five years. These technologies allow us to create microfluidic devices and peripherals. Some groups have reported 3D-printed microfluidic devices [24,25,26], and others have applied 3D printers for bioprinting [27,28]. The Lewis group fabricated microfluidic print-heads for 3D printing [29]. 3D printers can save labor for the first stage device fabrication and reduce fabrication limitation in the field of microfluidics. On the other hand, although 3D printing is a powerful technique for rapid prototyping, personalized manufacturing, and distributed manufacturing, it is still less efficient than injection molding and other conventional techniques from the viewpoint of mass production.

In recent years, thiol-ene reaction has attracted a lot of attention as an alternative to poly(methyl methacrylate) (PMMA) or poly(dimethylsiloxane) (PDMS) [30,31,32,33,34]. The thiol-ene reaction-based materials polymerize rapidly, have solvent resistance, have good mechanical properties, and have the possible application of 3D fabrication. We also applied the reaction to fabricate a thermo-pneumatic pump that is composed of several layers [35]. Carlborg *et al.* reported a new thiol-ene reaction-based microfabrication [36]. They characterize mechanical and chemical properties of microfluidic devices based on the thiol-ene reaction and demonstrate a potential to bridge the gap between rapid prototyping and mass production. However, their fabrication technique is limited to 2D-designed microfluidic channels. In this study, we propose a simple rapid prototyping method based on soft lithography without any support material for high throughput 3D fabrication. The method is a combination of injection-molding and lamination, which uses double-sided molding with PDMS molds and a UV-curable adhesive based on thiol-ene reaction. It can be done by conventional equipment for soft lithography and requires no cumbersome bonding and washing processes for multilayer lamination.

## 2. Materials and Methods

### 2.1. Device Fabrication

**Figure 1:**
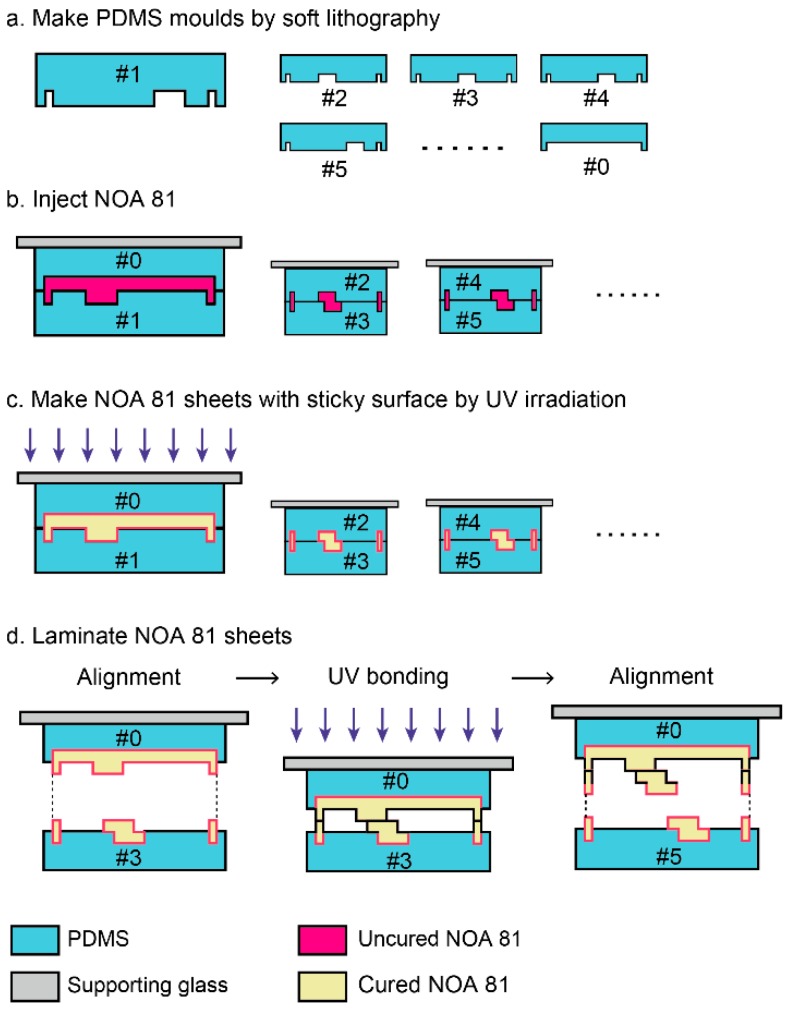
Schematic cross-sectional illustrations of the 3D fabrication process by conventional photolithography with NOA 81. (**a**) Fabricated PDMS molds by soft lithography. The numbers indicate layer order and #0 is a mold for a lid. (**b**) Injection of uncured NOA 81 to the spaces between PDMS molds by capillary force. (**c**) UV irradiation for partially curing NOA 81. (**d**) Lamination of NOA 81 sheets. NOA sheets are aligned after one side of PDMS molds are peeled off. The sheets are bonded by UV irradiation. The alignment and UV bonding processes are repeated.

The fabrication process is schematically depicted in Figure 1. There are two main procedures in the fabrication method: the fabrication of PDMS molds by soft lithography and the lamination of thin sheets. The PDMS molds are used for making thin sheets of a commercial UV-curable adhesive, Norland Optical Adhesive 81 (NOA 81). NOA 81 can be cured rapidly by UV irradiation within a few minutes or even seconds. However, the reaction is inhibited by oxygen, and PDMS has high oxygen permeability and UV transmittance. The NOA 81 in the PDMS molds is cured by UV irradiation, while surfaces of NOA 81 sheets in contact with PDMS remains uncured [31,33]. The uncured surfaces work as bonding layers, which are helpful in piling up thin layers to 3D structures.

The sliced images of objective structures along the *Z*-axis were drawn on overhead projector (OHP) films that were used as photomasks. The master molds were made with negative photoresist SU-8 series (Microchem, Tokyo, Japan) to make PDMS molds. Two components of the PDMS kit (Sylgard 184, Dow Corning, Tokyo, Japan) were mixed at a weight ratio of base:curing agent = 10:1. The prepolymer was poured into the SU-8 master molds. Concavities and convexities of the PDMS molds were opposite to those of conventional PDMS microfluidic channels; channels in SU-8 molds were concaves, and channels in PDMS molds were convexes (Figure 1a).

The PDMS molds of the objective 3D structure were divided into pairs to make interspaces. The upper PDMS molds were attached to supporting glass plates such as a cover glass or a glass slide for preventing the upper PDMS molds from dead load deflection. The interspaces were filled with NOA 81 (Norland Products Inc., Cranbury, NJ, USA) by capillary force (Figure 1b). Uncured NOA 81 were polymerized by UV irradiation for 90 s with a simple black light (AS ONE Co., Osaka, Japan) that illuminates 1.65 mW/cm^2^ at 365 nm. As mentioned above, the polymerized NOA 81 sheets with a cross-sectional shape of the objective 3D structure have uncured surfaces (Figure 1c). The NOA sheets were bonded via UV irradiation for 30 s after one side of the PDMS molds were peeled off (Figure 1d). The lamination process was repeated until the top layer. Thin films were aligned with a positioning stage (TR6047-S1, Chuo Precision Industrial Co. Ltd., Tokyo, Japan) under a stereomicroscope (SZ61, Olympus, Tokyo, Japan). The devices with 3D structures were exposed to UV light for 20 min for complete polymerization, because NOA 81 requires 2 J/cm^2^ to fully cure, according to the product data sheet. Three kinds of 3D structures—Menger sponges, spiral structures, and a channel in channel structure—were fabricated by using this method.

### 2.2. Bond Strength Measurement

Bond strength between NOA 81 sheets was measured by a tensile adhesion test with glass slides bonded by NOA 81 sheets. Two PDMS molds without any pattern were put on two glass slides, and uncured NOA 81 was injected into the spaces between the molds and the glass slides. Two glass slides with NOA 81 were exposed to UV light for 90 s. The NOA 81 sheets were cut into 1 × 1 cm^2^ with a utility knife after the PDMS molds were peeled off from glass slides. The two glass slides were bonded with the NOA 81 by UV exposure for 20 min. A weight was hung on a glass slide, and the load was made heavier to reach the point at which the bonding between NOA 81 sheets broke.

### 2.3. Flow Visualization

The 3D microstructures and 3D fluidics in the channels were observed with a scanning electron microscope (SEM, TM-1000, Hitachi, Tokyo, Japan) and a laser confocal microscope (TCS-STED-CW, Leica Microsystems, Wetzlar, Germany). Aqueous solutions of 0.5 mM fluorescein sodium salt (Sigma-Aldrich, Tokyo, Japan) and 0.1 mM rhodamine B (Sigma-Aldrich, Tokyo, Japan) were used to visualize the 3D flow and observed with a 442-nm excitation laser and a 10×/0.40 lens (HCX PL APO CS, Leica Microsystems GmbH, Wetslar, Germany). Stacks of each confocal X-Y scan of 1024 × 1024 pixels were collected with a step of 0.49 µm in the Z direction. Z-series images were loaded in to the imaging software (LAS AF, Leica Microsystems GmbH, Wetslar, Germany) and made into vertical cross-sectional images.

## 3. Results and Discussion

### 3.1. Characterization of The Method

**Figure 2:**
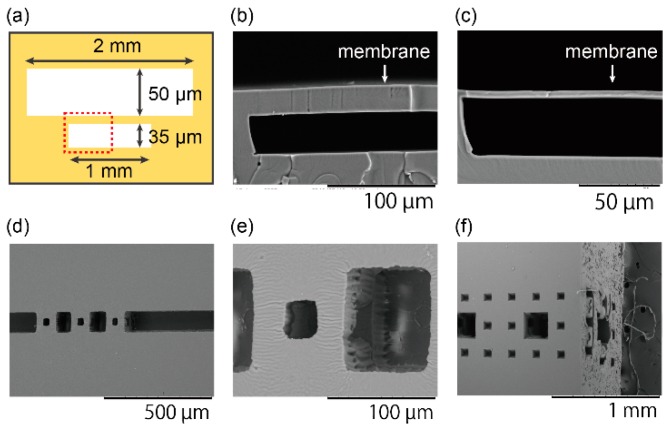
Structures for characterization. (**a**) A schematic illustration of a structure for layer thickness characterization. A red dotted box shows a region for close-up views in (**b**,**c**). (**b**) An SEM image of a membrane made by a PDMS mold with a thickness of 50 µm and (**c**) a membrane made by a PDMS mold with a thickness of 4 µm. (**d**) 90-µm level-1 Menger sponges; (**e**) A close up image of the level-1 Menger sponge. (**f**) 810-µm level-2 Menger sponges from oblique view points.

Pneumatic valve-like structures, which have a membrane clamped with 35-µm-deep and 50-µm-deep chambers, were fabricated to confirm the minimum layer thickness (Figure 2a). Figure 2b,c shows the SEM images of cross-sectional shapes of the membranes. SU-8 3025 was used to make a PDMS mold for a thicker membrane, and SU-8 3005 was used for a thinner membrane. The layer thickness can be controlled by changing the thickness of the SU-8 master molds, and the minimum layer thickness was 4 µm. This performance is higher than that of the high-end commercialized 3D printer with a minimum layer resolution of around 10 µm [26,37,38,39,40,41].

As for the bond strength, two pieces of NOA 81 sheets could maintain the bond against a load of 3.4 kg for a few minutes. The bond between NOA 81 sheets was broken upon loading 3.5 kg. The bond strength with a bonding area of 1 cm^2^ against a load of 3.4 kg was about 330 kPa. The average bond strength of the conventional bonding method was from 100 to 500 kPa [42,43,44,45]. The bonding between NOA 81 sheets by our method has enough bond strength for using it as a material in microfluidic devices.

The Menger sponge is one of the fractals that are geometric configurations with a self-similar pattern [46]. The basic structure is a cube with holes on every surface in the center. The 20 basic structures are arranged in a way that they form the same configuration. The higher level Menger sponges are made by the Menger sponges of the same regulation one level lower. In this paper, level-1 and level-2 Menger sponges were fabricated. Figure 2d–f shows SEM images of 90-µm level-1 and 810-µm level-2 Menger sponges. They are composed of 30-µm cubes and 270-µm level-1 Menger sponges, respectively. The alignment accuracy is less than 10% of the minimum structure size; however, the accuracy can improve by using bonding aligner. Their unique structure with many undercuts can be observed.

### 3.2. Spiral Structure

**Figure 3:**
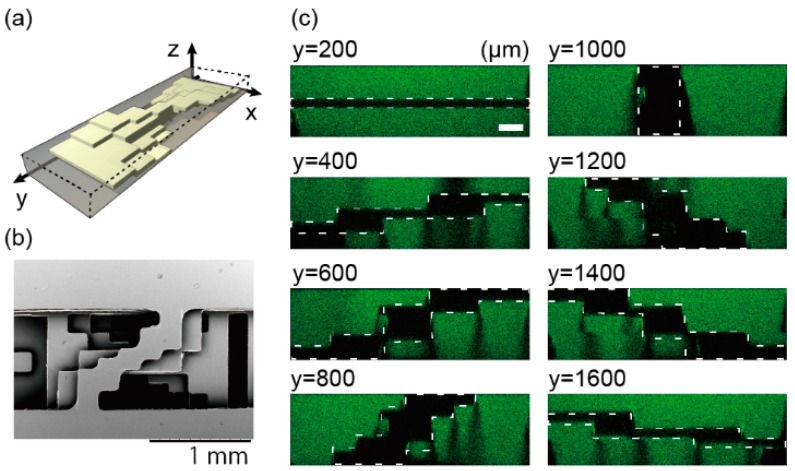
Diagrams of a spiral structure. (**a**) A conceptual image of the structure. (**b**) A SEM image of the structure from top-down view. (**c**) Confocal images of vertical cross sections at every 200 µm in the flow direction of the microchannel filled with fluorescein solution. White dotted lines represent cross-sectional shapes of a five-layer structure. Scale bar is 100 µm.

The spiral stair-like structure has a deforming wall in a channel, as shown in Figure 3a. This wall consists of five layers, and its cross sections change their shape to rotate 180° around a central longitudinal axis of a channel. Figure 3b,c is the top-down view of the spiral structure taken with a SEM and cross-sectional shapes taken with a confocal microscope, respectively. The structure, consisting of five thin NOA 81 films (black parts), shows the changing cross-sectional shapes: a horizontal wall appears at 200 µm from the channel end, changes to a vertical wall at the point of 1000 µm, and the vertical wall keeps rotating until it becomes horizontal at 1800 µm. The thickness of the NOA 81 sheets made with two molds were 119 ± 2.03 µm measured with a digital micrometer (CLM1-15QM, Mitsutoyo Corporation, Kanagawa, Japan) when the sheets were made in 60-µm-high SU-8 molds. The change of the structure shape can be smoother by a redesign of the structure (Movie S1) and an increase in lamination layers.

**Figure 4:**
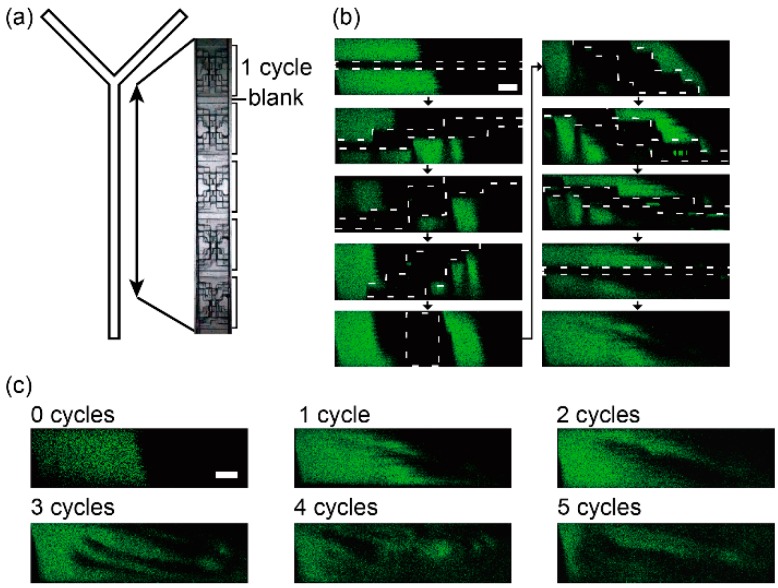
Confocal microscope images of a flow in the spiral structure channel. (**a**) Overhead view of the Y-shaped channel with the 5 spiral structures; (**b**) Confocal microscope images of vertical cross sections of the microchannel at every 200 µm in the flow direction and (**c**) at blanks. The channel was filled with a fluorescein solution (green) and water (dark). Scale bars are 100 µm.

The spiral structures were embedded in a Y-junction microfluidic channel to visualize the flow in the microchannel (Figure 4a). One cycle of the structure was 2 mm in length and there were five cycles in the channel. Fluorescein solution and water were introduced into the channel with syringe pumps (KD Scientific, Holliston, MA, USA) and the flow in the channel was observed with the confocal microscope. The fluids were mixed with the structure and the flow in the channel is similar to the flow in a chaotic microfluidic mixer [13,14] or a spiral type mixer [18] (Figure 4b,c). The two kinds of liquids were rotated by the change in cross-section structure and they generated a vertical multilayer flow. The multilayer spiral structure has a potential to make and control the 3D flow.

**Figure 5:**
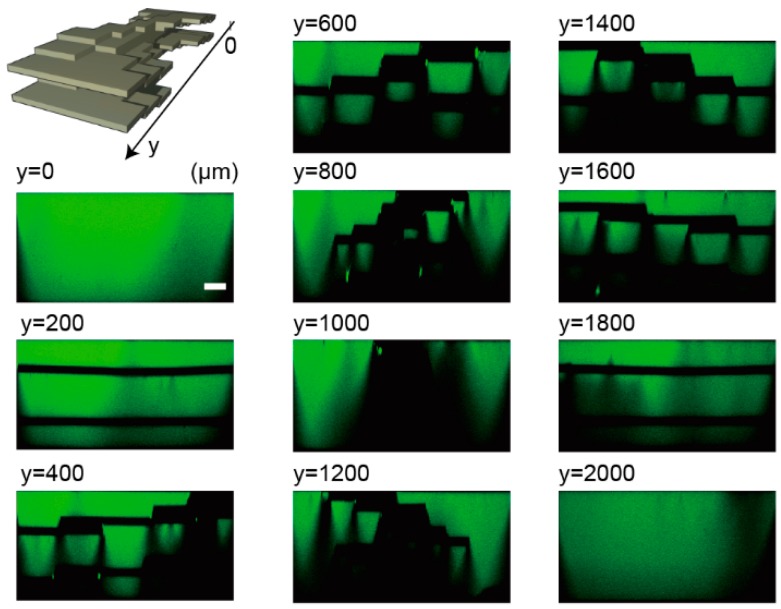
Confocal microscope images of a ten-layer spiral structure at every 200 µm in the flow direction of the microchannel. Scale bar is 100 µm.

We also made a ten-layer spiral structure in a microfluidic channel, which has two five-layer spiral structures in the channel (Figure 5). Although the lower layer cannot be visualized clearly due to light scattering at the vertical shape of the structure, the double screw structure was observed. The structure has an extruded structure from side to side and from top to bottom, which cannot be fabricated by conventional soft lithography. When the channel was pressurized to introduce a fluorescent reagent with the syringe pump (flow rate: 1 mL/min), there was no liquid leakage from the channel. The same PDMS molds can make another type of ten-layer structure made of the upper and lower five-layer 3D structures.

3.3. 3D Sheath Device

**Figure 6:**
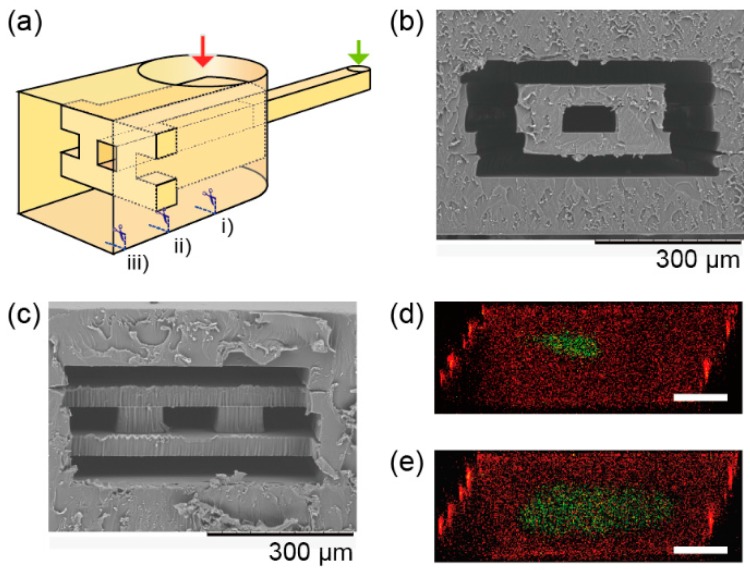
Cross-sectional images of a 3D sheath device. (**a**) Conceptual image of the 3D sheath device. (**b**) A SEM image of a cross section of the device at position (i) in Figure 6a, and (**c**) at position (ii) in Figure 6c. (**d**) A confocal microscope image of the 3D sheath at position (iii) with the flow rate of fluorescein as 90 µL/min and rhodamine as 450 µL/min. (**e**) Fluorescein as 40 µL/min and rhodamine as 210 µL/min. Scale bars are 100 µm.

A 3D sheath device was also made via the fabrication method. The 3D sheath device has a channel with a smaller channel in it, and the channels are connected to different inlets (Figure 6a). The smaller channel is merged with a larger channel to make a sheath flow (Figure 6b,c). The larger channel dimension is 500-µm-wide and 250-µm-deep, and the smaller one is 100-µm-wide and 50-µm-deep. A flow in the merged channel was observed with the confocal microscope at 5 mm from the merged point while a fluorescein solution and rhodamine solution were introduced into the channels from the two inlets with the flow rate of fluorescein as 90 µL/min and rhodamine as 450 µL/min. The fluorescein solution was stably sheathed by the rhodamine solution at least 5 mm in length. The sheathed flow broadened due to decreasing flow rates—fluorescein as 40 µL/min and rhodamine as 210 µL/min.

## 4. Conclusions

In this paper, we demonstrated a 3D microfabrication method with simple instruments for soft lithography: a black light for detection of biomolecules and a stereomicroscope. The method does not require any robotic action or sacrificial materials [47,48]. The method exhibited enough bond strength for microfluidic devices and thin layer thickness with a high layer resolution. The Menger sponge-like structures, and some 3D microfluidic devices were achieved without limitation by the demolding process needed in conventional molding fabrication. This method still has some problems about the positioning accuracy for manual procedures, which can be solved by instruments and automation techniques for mass production. 3D structures in microfluidic channels grow in importance as approaches to control flow [49,50], produce microlens arrays [51], produce 3D cell culture systems and mimic organs [52,53,54,55], and develop multifactor separation [56]. Our fabrication method can meet the requirements of new 3D structures in a lab scale and allow for the commercialization of previously studied prototype devices.

## Supplementary Materials

The following are available online at http://www.mdpi.com/2072-666X/7/5/82/s1. Movie S1: The smooth shape change of the five-layer screw structure in a vertical cross section.
